# Cell wall composition and penetration resistance against the fungal pathogen *Colletotrichum higginsianum* are affected by impaired starch turnover in Arabidopsis mutants

**DOI:** 10.1093/jxb/erw434

**Published:** 2016-12-12

**Authors:** Timo Engelsdorf, Cornelia Will, Jörg Hofmann, Christine Schmitt, Brian B. Merritt, Leonie Rieger, Marc S. Frenger, André Marschall, Rochus B. Franke, Sivakumar Pattathil, Lars M. Voll

**Affiliations:** 1 Friedrich-Alexander-Universität Erlangen-Nürnberg, Division of Biochemistry, Staudtstrasse 5, D-91058 Erlangen, Germany; 2 Complex Carbohydrate Research Center, The University of Georgia, 315 Riverbend Road, Athens, GA 30602, USA; 3 Universität Bonn, Institute for Cellular and Molecular Botany, Department of Ecophysiology, Kirschallee 1, D-53115 Bonn, Germany; 4 Technische Hochschule Nürnberg Georg-Simon Ohm, D-90121 Nürnberg, Germany

**Keywords:** Carbohydrate metabolism, cell wall, glycome profiling, hemibiotrophy, host–pathogen interaction, indolic glucosinolates, pectin, penetration resistance, plant defense, starch deficiency.

## Abstract

Penetration resistance represents the first level of plant defense against phytopathogenic fungi. Here, we report that the starch-deficient *Arabidopsis thaliana phosphoglucomutase* (*pgm*) mutant has impaired penetration resistance against the hemibiotrophic fungus *Colletotrichum higginsianum.* We could not determine any changes in leaf cutin and epicuticular wax composition or indolic glucosinolate levels, but detected complex alterations in the cell wall monosaccharide composition of *pgm*. Notably, other mutants deficient in starch biosynthesis (*adg1*) or mobilization (*sex1*) had similarly affected cell wall composition and penetration resistance. Glycome profiling analysis showed that both overall cell wall polysaccharide extractability and relative extractability of specific pectin and xylan epitopes were affected in *pgm*, suggesting extensive structural changes in *pgm* cell walls. Screening of mutants with alterations in content or modification of specific cell wall monosaccharides indicated an important function of pectic polymers for penetration resistance and hyphal growth of *C. higginsianum* during the biotrophic interaction phase. While mutants with affected pectic rhamnogalacturonan-I (*mur8*) were hypersusceptible, penetration frequency and morphology of fungal hyphae were impaired on *pmr5 pmr6* mutants with increased pectin levels. Our results reveal a strong impact of starch metabolism on cell wall composition and suggest a link between carbohydrate availability, cell wall pectin and penetration resistance.

## Highlight

Changes in Arabidopsis cell wall composition, predominantly in pectic polymers, influence penetration and establishment of *Colletotrichum higginsianum* hyphae in host leaves during initial biotrophy.

## Introduction

Penetration of the host leaf tissue by phytopathogenic fungi can occur either through stomatal pores or by breaching epidermal cell walls by combined action of enzymatic degradation and physicomechanical force. The subsequent establishment of hyphae inside the host tissue enables the pathogen to access nutrients of the invaded host tissue. The plant cuticle and cell wall represent the first line of structural physicochemical barriers that oppose fungal penetration.

The cuticle represents the outermost hydrophobic layer of aerial plant organs and can roughly be divided into two distinct layers. The cuticle proper, containing the lipid polyester layer, cutin, is dominated by crosslinked hydroxylated C_16_ and C_18_ fatty acids with embedded intracuticular waxes. The cutin layer is covered by epicuticular wax crystals composed of very-long-chain (>C_20_) fatty acid derivatives (as reviewed by Beisson *et al.*, 2012; Nawrath *et al.*, 2013). The chemical composition of both cutin and wax was found to play an important role in plant–fungal interactions. In recent years it was demonstrated that particular hydroxylated fatty acids (Mendoza-Mendoza *et al.*, 2009), long-chain alcohols (Uppalapati *et al.*, 2012) or long-chain aldehydes (Reisige *et al.*, 2006; Hansjakob *et al*., 2010, 2011) in epicuticular wax are required for efficient spore differentiation and appressoria formation of (hemi)biotrophic fungal pathogens. On the other hand, cutin deficiencies can be associated with increased resistance of Arabidopsis to the fungal necrotroph *Botrytis cinerea*. Current models account an increased permeability of the cuticle to pathogen- or host-derived elicitors that are generated during cutin and cell wall degradation for a subsequently enhanced recognition by host cells (reviewed by Yeats and Rose, 2013). Nevertheless, contrasting correlations between cutin thickness and *B. cinerea* resistance have been obtained with other host plants.

Preinvasion resistance can also be accomplished by generating toxic compounds. Accumulation of the indolic glucosinolate (IG) 4-methoxy-indole-3-ylmethyl-glucosinolate (4MOI3M) and 4MOI3M cleavage by the atypical myrosinase PEN2 contribute to pre-invasion resistance against a wide range of fungal pathogens in Arabidopsis, including non-adapted powdery mildew fungi and *Colletotrichum* species (Lipka *et al.*, 2005; Bednarek *et al.*, 2009; Hiruma *et al.*, 2010). Penetration frequency of the hemibiotrophic ascomycete *C. higginsianum* is only slightly increased on *pen2* mutants, and therefore the role of IGs in the interaction with this adapted *Colletotrichum* species is as yet unclear (Huser *et al.*, 2009). Deposition of the β(1,3)-linked glucan callose in cell wall papillae formed at attempted fungal penetration sites has been demonstrated to effectively restrict powdery mildew penetration (Ellinger *et al.*, 2013). However, formation of callose papillae did not substantially contribute to penetration resistance against *Colletotrichum higginsianum* (Narusaka *et al.*, 2004; Shimada *et al.*, 2006; Birker *et al.*, 2009; Huser *et al.*, 2009).

In addition to the previously mentioned physical barriers, successful fungal penetration pegs need to penetrate the plant cell wall, a complex hydrated matrix of polysaccharides and proteins that confer mechanical stability on plant cells. In Arabidopsis, the water-insoluble scaffold of primary cell walls in expanding leaves is composed of about 14% cellulose, 24% hemicellulose and 42% pectin (Zablackis *et al.*, 1995). Hemicelluloses are composed of neutral sugar moieties that form β(1,4)-linked polysaccharide backbones, with xyloglucan being the predominant type in primary Arabidopsis walls (Scheller and Ulvskov, 2010). Negatively charged galacturonic acid (GalA) residues are abundant in the pectic polymers xylogalacturonan, rhamnogalacturonan-I, and rhamnogalacturonan-II, while homogalacturonan is exclusively composed of GalA (Harholt *et al.*, 2010). Hemicellulose and pectin polymers are thought to form a highly crosslinked matrix that is linked to the scaffold-forming cellulose microfibrils and plays a central role in the regulation of cell wall elasticity and rigidity (for current models on cell wall polymer interconnection, see reviews by Cosgrove and Jarvis, 2012; Peaucelle *et al.*, 2012). The degree of pectin methylesterification (DM) is controlled by pectin methylesterase (PME) and PME inhibitor (PMEI) function and controls cell wall stiffness as demonstrated by atomic force microscopy (Peaucelle *et al*., 2011, 2015). However, seemingly contradictory reports exist on the effect of DM on cell wall stiffness and cell elongation, suggesting cell-specific differences (reviewed by Levesque-Tremblay *et al.*, 2015).

Both the structural composition of the cell wall matrix and the modification of cell wall polymers have been identified as compatibility factors during the interaction of plants with fungal biotrophs and necrotrophs. Penetration pegs of most biotrophs induce limited hydrolysis of cell wall polymers. Carbohydrate-active enzyme families are expanded in fungal hemibiotrophs and necrotrophs that rely on the secretion of hydrolases to access the cell wall as a complex carbon source (e.g. Ma *et al.*, 2010; O’Connell *et al.*, 2012). Increased resistance of the Arabidopsis *powdery mildew resistant 5 (pmr5*) and *pmr6* mutants towards the adapted powdery mildew fungus *Golovinomyces cichoracearum* is associated with increased pectin content of the cell walls (Vogel *et al.*, 2002, 2004). *PMR6* has been identified as encoding a pectate lyase-like protein (Vogel *et al.*, 2002), while PMR5 has been characterized as a trichome birefringerence-like (TBL) protein (TBL44) that is likely to affect cell wall *O*-acetylation (Gille and Pauly, 2012). Congruently, reduced *O*-acetylation in the Arabidopsis mutant *reduced wall acetylation 2* (*rwa2*) or Arabidopsis plants overexpressing fungal acetylesterase was associated with increased resistance to the fungal necrotroph *Botrytis cinerea* (Manabe *et al.*, 2011; Pogorelko *et al.*, 2013). There is evidence that pectinolysis by fungal polygalacturonases (PGs) plays an important role during pathogenesis of fungal necrotrophs. For instance, antisense repression of a host-borne PG inhibitor protein resulted in increased susceptibility of Arabidopsis towards *B. cinerea* (Ferrari *et al.*, 2006). In turn, transgenic expression of a fungal PG strongly induced host defense responses in *B. cinerea* infected leaves (Ferrari *et al.*, 2008). This might be explained with an elevated release of host-derived damage-associated molecular patterns (DAMPs) like oligogalacturonides and their subsequent recognition by wall-associated kinases that act as DAMP receptors (Ferrari *et al.*, 2007; Kohorn and Kohorn, 2012). Arabidopsis plants with increased PMEI activity or reduced PME activity exhibited an increased resistance to *B. cinerea*, suggesting that a high DM either reduces pectin accessibility for PG degradation or interferes with fungal penetration through altered cell wall stiffness (Lionetti *et al.*, 2007; Raiola *et al.*, 2011).

*Colletotrichum higginsianum* is a hemibiotrophic ascomycete fungus adapted to the model plant *Arabidopsis thaliana* (for reviews on the life style of *Colletotrichum spp.* see Mendgen and Hahn, 2002; Münch *et al.*, 2008). Leaf infections with *C. higginsianum* are initiated by the germination of conidia on the leaf surface. At the tip of the germ tubes, dome-shaped melanized appressoria differentiate; they accumulate sugar alcohols and build up a high turgor pressure upon the subsequent diffusion of water from outside water droplets into the appressoria. The wall of the underlying epidermal cell is subsequently pierced by a penetration peg with a combination of mechanical force and lytic enzyme activity (Bechinger *et al.*, 1999; Deising *et al.*, 2000). In the penetrated host epidermis cells, *C. higginsianum* establishes itself within 36 h post-inoculation by forming a bulbous infection vesicle that produces lobed biotrophic primary hyphae. At around 72 h post-inoculation, neighboring cells are colonized by rapidly growing, narrow-bore necrotrophic secondary hyphae, which leads to visible necrotic lesions on infected leaves.

Recently, we have shown that reduced diurnal carbon availability in genotypes with reduced starch or carboxylate turnover leads to increased susceptibility of Arabidopsis toward *C. higginsianum*. In addition, we observed a connection between carbon shortage and delayed defense responses in host leaves, which had most deleterious consequences during necrotrophic growth of the pathogen (Engelsdorf *et al.*, 2013). In the present report, we demonstrate that Arabidopsis mutants with deficient starch biosynthesis or mobilization show faster penetration and establishment of biotrophic hyphae inside of host cells. We reveal complex alterations in the cell wall composition of starch-deficient *phosphoglucomutase* (*pgm*) mutants and present evidence that pectin quality and quantity can influence penetration and biotrophic establishment of *C. higginsianum* in Arabidopsis.

## Materials and methods

### Plant and fungal material and growth conditions

Arabidopsis plants were grown as described in Engelsdorf *et al.* (2013). Seeds for *pgm* (N210), *adg1-1* (N3094), *sex1-1* (N3093), *mur4-1* (N8568), *mur8-1* (N8575), *mur11-1* (N8579), *arad1-2* (N25046), *gals1-1* (N657519), *pmr5* (N6579), *pmr6-3* (N66990), *pmr5 pmr6-3* (N6580), *pme3-1* (N400106), *pme35-1* (N661699), and *pmei6-2* (N475768) were obtained from Nottingham Arabidopsis Stock Centre (NASC; University of Nottingham, UK). *hig1-1*, *myb34/myb51* and *HIG1-1D* seeds were kindly provided by Tamara Gigolashvili (Institute of Botany, University of Cologne; Gigolashvili *et al.*, 2007; Frerigmann and Gigolashvili, 2014). All mutants and transformants were in the Col-0 background.

*Colletotrichum higginsianum* isolate MAFF 305635 (Ministry of Agriculture, Forestry and Fisheries, Japan) was grown on oat meal agar plates (5% (w/v) shredded oat meal, 1.2% (w/v) agar) for 7 d at 22 °C under illumination to promote conidia formation.

### *C. higginsianum* infection assays

Leaf infection by *C. higginsianum* was performed by spray inoculation with a conidia titer of 2 × 10^6^ conidia ml^–1^ as described by Voll *et al.* (2012).

### Assessment of *C. higginsianum in planta* development and evaluation of susceptibility

Fungal structures were stained using trypan blue as described in Koch and Slusarenko (1990). Microscopy was performed on a Leica DMR microscope (Bensheim, Germany) with differential interference contrast optics.

Quantification of the relative genomic *C. higginsianum* DNA content was performed as previously described (Engelsdorf *et al.*, 2013).

### Quantification of indolic glucosinolate content

Glucosinolates were extracted and purified as described by Gigolashvili *et al.* (2007). Separation of desulfoglucosinolates was performed on a Dionex Ultimate 3000 HPLC system (DGP-3600MB, WPS-3000TB, PDA-3000) equipped with a Phenomenex Luna Security Guard C18 column (4.0 × 3.0 mm) and a Luna C18(2) reverse-phase column (5 µm, 250 × 4.6 mm) at 25 °C column temperature and a flow rate of 1 ml min^−1^ using the following gradient: 0–5 min, 0% acetonitrile (ACN); 5–30 min, 30% ACN; 30–32 min, 40% ACN; 32–36 min, 40% ACN; 36–40 min, 0% ACN; 40–50 min, 0% ACN. Peaks were quantified at 229 nm relative to an internal benzyl glucosinolate standard using the respective response factors described by Brown *et al.* (2003).

### Analysis of epicuticular wax and cutin

For wax analysis 10–20 rosette leaves (corresponding to 10–15 cm^2^) of 5-week-old plants were cut and immediately immersed in chloroform for 10 s at room temperature. The resulting solution containing the cuticular waxes was spiked with 10 µg of tetracosane (Fluka) as an internal standard. The solvent was evaporated under a stream of nitrogen, and compounds containing free hydroxyl and carboxyl groups were converted into their trimethylsilyl ethers and esters, respectively, with bis-(*N*,*N*-trimethylsilyl)-trifluoroacetamide (Machery-Nagel) in pyridine for 40 min at 70 °C before GC-MS analysis. Wax constituents were identified by their electron-impact MS spectra after GC-MS analysis and quantified using an identical GC system equipped with a flame ionization detector (FID) as described previously (Kurdyukov *et al.*, 2006).

For cuticular leaf polyester composition, wax extracted leaves were then exhaustively extracted with chloroform–methanol (1:1; v/v) with daily change of solvent over a period of 7 d to remove soluble lipids. Cutin monomers were solubilized by transesterification with 1 M methanol/HCl (Supelco) for 2 h at 80 °C in a Teflon-sealed screw-cap tube. After addition of 2 ml saturated NaCl/H_2_O the hydrophobic monomers were subsequently extracted three times in hexane containing 10 µg of the internal standard dotriacontane. The combined extracts were evaporated and derivatized as described above and subsequently analysed by GC-MS and GC-FID as described previously (Franke *et al.*, 2005).

### Toluidine blue staining

Toluidine blue staining was conducted as described by Bessire *et al.* (2007). Four droplets of a 0.025% toluidine solution were spotted onto each of nine fully expanded leaves from nine independent plants per genotype.

### Cell wall preparation and analysis

Starch-free alcohol-insoluble residue (AIR) for cellulose analysis was prepared from 50 mg of Arabidopsis leaf powder by extracting twice with 80% ethanol at 80 °C followed by incubation in 90% dimethyl sulfoxide–10% _dd_H_2_O for four times at 24 h at room temperature (RT). AIR was then washed three times with _dd_H_2_O and two times with acetone. Subsequently, dried AIR was treated with 5 U of α-amylase (Sigma-Aldrich A3176) in 100 mM ammonium formate for 72 h and washed as above. Cellulose content of dried starch-free AIR was determined spectrophotometrically following the method of Updegraff (1969) and quantified in relation to glucose standards.

For the analysis of cell wall monosaccharide composition and enzymatic digestions of cell wall preparations, 30–40 mg of crushed leaves was extracted twice with 1 ml of 80% ethanol at 80 °C for 20 min and once with 1 ml acetone at RT for 5 min. Extracted leaves, i.e. crude cell wall preparations, were dried at 60°C for 10 min.

For digestion of crude cell wall preparations with arabinanase, 2 U of endo-arabinanase (Prod. Code E-EARAB, Megazyme, Bray, Ireland) in 1.4 ml 50 mM sodium acetate (pH 5.5) was added to dried cell wall pellets and the samples were incubated for 12 h at 37 °C. The reactions were stopped by the addition of 650 µl cold 95% ethanol–10 mM EDTA and the digested cell wall material was sedimented by centrifugation for 5 min at 20 000 *g*. For galactanase digestion, the pH of the cell wall pellets was adjusted to 4.7 with 70 µl 0.1 M KOH and 2 U of endo-1,4-β-D-galactanase (Prod. Code E_EGALN, Megazyme) was added in 1.4 ml 100 mM acetic acid prior to incubation at 40 °C for 12 h.

Water-soluble polymers were isolated as described by Reiter *et al.* (1997) with minor modifications. Crude cell wall fractions were prepared and dried as described above and resuspended in 1 ml ice-cold buffer I (100 mM MOPS pH 7.0, 1.5% sodium dodecyl sulfate (SDS), 5 mM sodium bisulfite) with a rotating pestle. After sonication for 5 min in an ice-water bath (Bandelin Sonopuls HD 2070 and UW 2027, 50% duty cycle, 50% power), insoluble material was pelleted at 16 000 *g* and 4 °C for 10 min. The pellet was washed with 1 ml of ice-cold buffer II (100 mM MOPS pH 7.0, 0.5% SDS, 3 mM sodium bisulfite) and centrifuged as above. After combination of the supernatants and the soluble fraction, the pellet was washed once with 1 ml of buffer II, three times with water and once with 1 ml of 80% ethanol. To the soluble fraction, 8 M ammonium acetate was added to a final concentration of 1 M, followed by precipitation of polymers with 5 volumes of ethanol–acetone (1:1) for 16 h at RT and centrifugation at 3200 *g* for 15 min.

Cell wall pellets were hydrolysed and analysed as previously described (Øbro *et al*., 2004). Briefly, pellets were resuspended in 1 ml of 2 M trifluoroacetic acid with a rotating pestle and hydrolysed at 120 °C for 1 h. After centrifugation at 16 000 *g* for 2 min, the supernatant was dried under vacuum and resuspended in water. Neutral monosaccharides and galacturonic acid were analysed by high-performance anion exchange with pulsed amperometric detection with a Dionex ICS-3000 system equipped with a CarboPac® PA20 column. To obtain base line separation of xylose and mannose, elution was started isocratically with 100% buffer C (2 mM NaOH; Fluka 72064) for 21 min. In 2 min the eluent was changed to 5% buffer A (20 mM NaOH, 1 M sodium acetate), 50% buffer B (200 mM NaOH) and 45% buffer C, in the following 19 min to 30% buffer A, 50% buffer B and 20% buffer C and then in 5 min to 50% buffer A and 50% buffer B. After 2 min of isocratic elution, the eluent was changed to 100% buffer B in 2 min and kept isocratic for 3 min to wash the column. The eluent was then changed to 100% buffer C in 3 min, followed by 13 min of re-equilibration. To obtain base-line separation of rhamnose and arabinose, elution was started isocratically with 4% buffer B and 96% buffer C for 14 min. In 2 min the eluent was changed to 2% buffer A, 48% buffer B and 50% buffer C, in the following 16 min to 30% buffer A, 50% buffer B and 20% buffer A, and then in 4 min to 50% buffer A and 50% buffer B. Washing and re-equilibration of the column was performed as above.

### Glycome profiling

Cell wall extraction and glycome profiling analyses of cell wall material were carried out as previously described (Demartini *et al.*, 2011; Pattathil *et al.*, 2012). In brief, cell walls (AIR) were sequentially extracted with increasingly harsh reagents [50 mM ammonium oxalate, 50 mM sodium carbonate (with 0.5% sodium borohydride; pH 10), 1 M KOH (with 1% sodium borohydride) and 4 M KOH (with 1% sodium borohydride)] at a ratio of 2 ml per 10 mg of AIR. The extracts were enzyme-linked immunosorbent assay (ELISA) screened with a comprehensive suite of cell wall glycan directed monoclonal antibodies (mAbs) monitoring most major non-cellulosic plant glycans (Pattathil *et al.*, 2012). To conduct these ELISAs cell wall extracts were coated on an equal carbohydrate basis (15 μl from 20 μg ml^−1^ stocks per well). Plant cell wall glycan-directed mAbs were from laboratory stocks (CCRC, JIM and MAC series) at the Complex Carbohydrate Research Center (available through CarboSource Services; http://www.carbosource.net, last accessed 16 November 2016) or were obtained from BioSupplies (Australia) (BG1, LAMP). Supporting information on mAbs (Pattathil *et al.*, 2010) used in this study can be found in Supplementary Table S1 at *JXB* online and the WallMabDB (http://www.wallmabdb.net, last accessed 16 November 2016) that provides detailed information for each antibody.

### Determination of soluble sugars and starch

Soluble sugars and starch were extracted and analysed as previously described by Voll *et al.* (2003).

### Statistical analysis

Student’s *t*-test was performed with SigmaPlot 12 (Systat Software Inc., Chicago, IL, USA) after testing for normality (Shapiro–Wilk test) and equal variance.

## Results

### *In planta* establishment of *C. higginsianum* is accelerated in starch-deficient *pgm* mutants

**Fig. 1.**
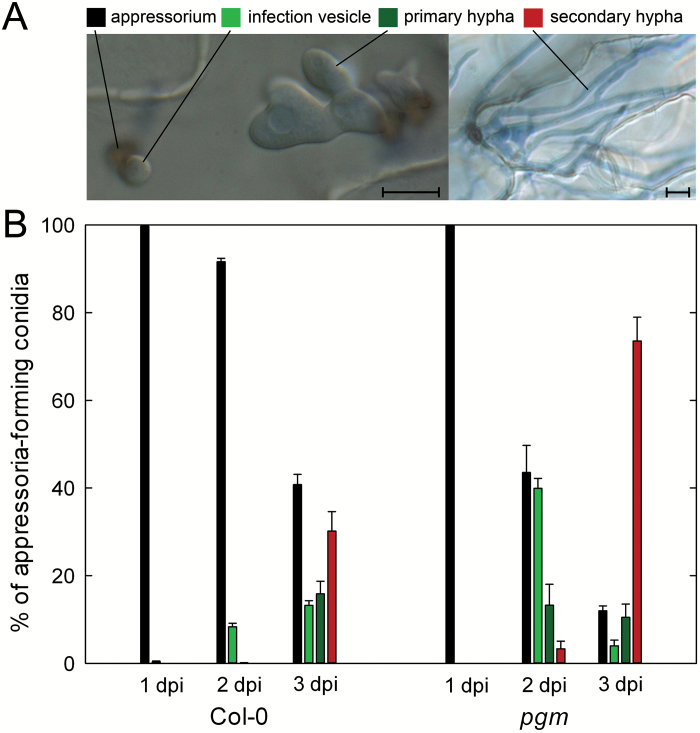
*Colletotrichum higginsianum* hyphae proliferation in *pgm.* Leaves of 5-week-old plants were infected with *C. higginsianum*, stained with trypan blue at 1, 2, and 3 d post infection (dpi) and examined by differential interference contrast microscopy. (A) Micrographs illustrating the scored fungal infection structures. Bars represent 10 µm. (B) Early fungal *in planta* development as given by the relative distribution of infection structures. Values are means of three biological replicates with four individual leaves each. Per replicate, the developmental status of *in planta* hyphae formed from 400–500 conidia was scored. The error bars represent the SE. Starting from appressoria, the most advanced infection structure was classified. The developmental order is as follows: appressoria, black bars; infection vesicles, light green bars; primary hyphae, dark green bars; secondary hyphae, red bars.

In order to analyse the impact of reduced diurnal carbohydrate availability on the early biotrophic interaction phase between Arabidopsis and *C. higginsianum*, fungal development was examined in leaves of the starch-deficient *phosphoglucomutase* (*pgm*) mutant. Compared with wild type, *pgm* mutant leaves exhibited a 10-fold accumulation of soluble sugars and showed an excessive export of sucrose from source leaves to roots (Engelsdorf *et al.*, 2013; Brauner *et al.*, 2014). This altered carbohydrate management leads to a 60% reduction in the diurnal turnover of carbohydrate storage in *pgm* source leaves and to periodic shortage for carbon towards the end of each dark phase (Gibon *et al.*, 2004; Engelsdorf *et al.*, 2013). Establishment of *C. higginsianum* was evaluated microscopically by scoring the most advanced fungal developmental stage visible at 1, 2, and 3 d post infection (dpi). After penetration by appressoria, infection vesicles (IVs) are formed first, followed by primary hyphae (PH) and secondary hyphae (SH) (Fig. 1A). At 1 dpi, appressoria had developed on both Col-0 and *pgm* leaves, while no fungal post-penetration structures could be observed in both genotypes. At 2 dpi, 8% of the assessed appressoria had successfully penetrated wild type leaves and differentiated IVs underneath the epidermal cell wall, whereas more than 55% of the appressoria on *pgm* had done so, giving rise to IVs (40%), PH (13%) and SH (3%) (Fig. 1B). Almost 80% of the appressoria on *pgm* leaves had formed SH at 3 dpi as compared with only 30% on wild type leaves (Fig. 1B). While the faster necrotrophic growth of *C. higginsianum* at 3 dpi can be explained by a reduction of induced defense responses in *pgm* leaves compared with wild type (Engelsdorf *et al.*, 2013), we aimed at understanding the cause of increased fungal colonization in *pgm* between 1 and 2 dpi.

### Cuticle composition is not affected in *pgm*

**Fig. 2.**
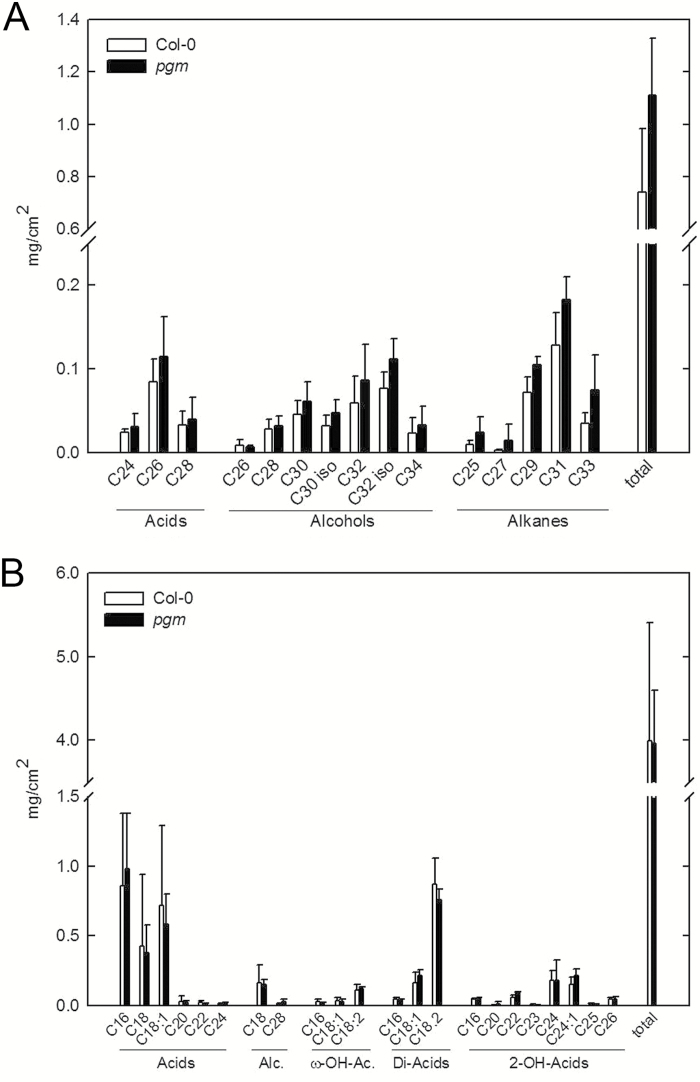
Epicuticular wax and cutin composition in *pgm.* Epicuticular wax (A) and cutin (B) monomer composition of fully expanded Col-0 (white bars) and *pgm* (black bars) leaves harvested from 5-week-old plants were determined in five biological replicates and are depicted as means ± SE. Wax constituents are shown as acids (left bracket), alcohols (middle bracket) and alkanes (right bracket). Cutin constitutents are abbreviated as follows: Alc., alcohols; ω-OH-Ac., ω-hydroxy acids; Di-Acids, dicarboxylic acids; 2-OH acids, 2-hydroxy acids. The chain lengths of the individual monomers are indicated below the bars, with the number behind the colon indicating the number of double bonds.

The epidermis represents the boundary tissue that gets in contact with pathogens first, and several studies have shown that the cuticle composition affects the outcome of plant–pathogen interactions (Yeats and Rose, 2013). We therefore analysed whether the composition of cuticular wax and/or cutin might account for the more rapid early development of *C. higginsianum* on *pgm* leaves. However, neither wax nor cutin composition was significantly altered in non-infected leaves of *pgm* mutants compared with wild type (Fig. 2). Consistently, toluidine blue staining, which indicates the permeability of the cuticle, was comparable between non-infected wild type (25.0 ± 7.2% of all inoculated sites were stained in nine independent leaves) and *pgm* leaves (27.8 ± 6.5% staining).

### Indolic glucosinolate levels do not affect penetration resistance against *C. higginsianum*

It has previously been shown, that indolic glucosinolates (IGs), especially the PEN2 substrate 4-methoxy-indole-3-ylmethyl-glucosinolate (4MOI3M), contribute to penetration resistance against non-adapted powdery mildew fungi and non-adapted *Colletotrichum* species in Arabidopsis (Bednarek *et al.*, 2009; Hiruma *et al.*, 2010), but the role of IGs during *C. higginsianum* attack is yet unclear.

**Fig. 3.**
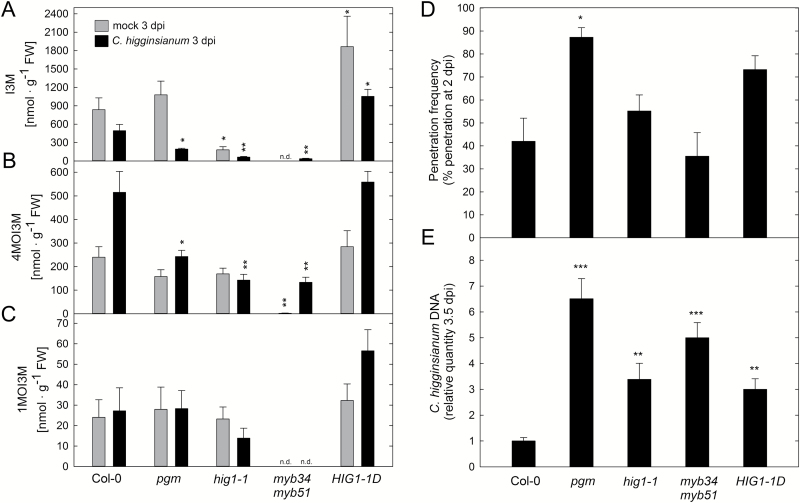
Analysis of indolic glucosinolate relevance in resistance to *C. higginsianum.* Indolic glucosinolate content was determined in 5-week-old leaves of Col-0, *pgm*, *hig1-1*, *myb34 myb51*, and *HIG1-1D* 3 dpi with *C. higginsianum* and mock treatment. (A–C) Indole-3-ylmethyl-glucosinolate (I3M; A), 4-methoxy-indole-3-ylmethyl-glucosinolate (4MOI3M; B), and 1-methoxy-indole-3-ylmethyl-glucosinolate (1MOI3M; C) contents were quantified in four biological replicates ±SE. (D) Penetration frequency as measured by the percentage of successful penetration events at 2 dpi. Values are means of three biological replicates ±SE. Per replicate, the penetration success of 200–300 appressoria on two leaves was scored. (E) The relative quantity of genomic *C. higginsianum* DNA in leaf samples was determined by qPCR at 3.5 d post-infection. Values are means of three biological replicates measured in three technical replicates ±SE. For each replicate, fully expanded leaves of two infected plants were pooled. Asterisks indicate a significant difference to Col-0 (*P<0.05; **P<0.01; ***P<0.001; Student’s *t*-test). n.d.: not detectable.

We observed that 4MOI3M contents were increased two-fold upon infection of Arabidopsis wild type with *C. higginsianum* (Fig. 3B). Furthermore, both indol-3-ylmethyl-glucosinolate (I3M) and 4MOI3M contents were significantly reduced in infected *pgm* mutant leaves compared with wild type, which prompted us to assess whether IG quantity can influence the Arabidopsis–*C. higginsianum* interaction. For this purpose, we analysed fungal proliferation in leaves of Arabidopsis genotypes with reduced (*high indolic glucosinolate 1-1*, *hig1-1/myb51*; *myb34 myb51*) and increased (*HIG1-1 DOMINANT*, *HIG1-1D*) IG content (Fig. 3A–C; Gigolashvili *et al.*, 2007; Frerigmann and Gigolashvili, 2014). Penetration frequency at 2 dpi was not significantly altered in *hig1-1*, *myb34 myb51* or *HIG1-1D* (Fig. 3D; with *P*=0.06 for *HIG1-1D*), indicating that reduced IG levels do not compromise preinvasion defense against *C. higginsianum*. During the necrotrophic phase at 3.5 dpi, however, fungal growth was increased in mutants with both reduced and increased IG content (Fig. 3E), suggesting that disturbance of IG metabolism interferes with postinvasion defense after fungal establishment is completed.

### Cell wall monosaccharide composition and penetration resistance are affected in Arabidopsis mutants with impaired starch metabolism

**Fig. 4.**
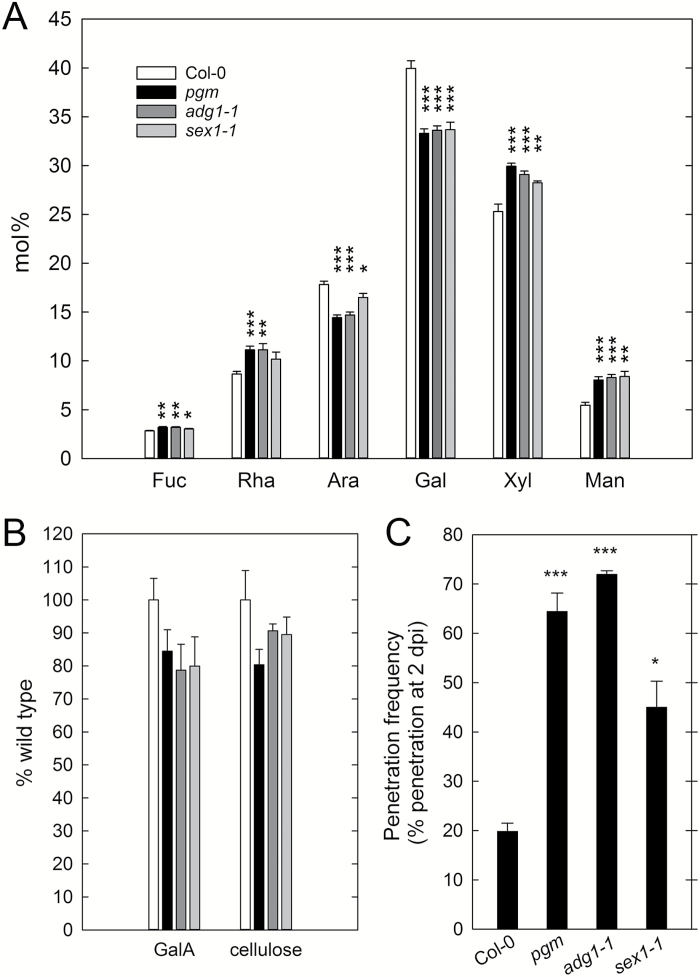
Cell wall monosaccharide composition and penetration resistance of Arabidopsis mutants with impaired starch metabolism. (A) Relative content (mol%) of the neutral monosaccharides fucose (Fuc), rhamnose (Rha), arabinose (Ara), galactose (Gal), xylose (Xyl) and mannose (Man) in 5-week-old rosette leaves of Col-0, *pgm*, *adg1-1* and *sex1-1*. (B) Galacturonic acid (GalA) and cellulose content in 5-week-old rosette leaves of Col-0, *pgm*, *adg1-1* and *sex1-1*. Col-0, white bars; *pgm*, black bars; *adg1-1*, dark grey bars; *sex1-1*, light grey bars. Values are means ±SE (*n*=6). (C) Penetration frequency of *C. higginsianum* was analysed at 2 dpi in 5-week-old Col-0, *pgm*, *adg1-1* and *sex1-1* leaves. Values are means of three biological replicates ±SE. Per replicate, penetration success of hyphae formed from 260–350 conidia on two leaves was scored. Asterisks indicate a significant difference from Col-0 (*P<0.05; **P<0.01; ***P<0.001; Student’s *t*-test).

Underneath the cuticle, the plant cell wall forms the second structural barrier for fungal penetration. It has previously been shown that susceptibility to fungal biotrophs and necrotrophs is also influenced by the composition and modification of host cell walls (Vogel *et al*., 2002, 2004; Hernández-Blanco *et al.*, 2007; Raiola *et al.*, 2011; Pogorelko *et al.*, 2013). We analysed the cell wall monosaccharide composition of *pgm* in comparison with the wild type. In fully expanded *pgm* leaves, we observed significantly reduced amounts of arabinose and galactose, while fucose, rhamnose, xylose, and mannose amounts were significantly increased compared with the wild type (Fig. 4A). Cell wall monosaccharide composition was similar in *pgm* and another starch-deficient mutant (*ADP-glucose pyrophosphorylase 1-1*, *adg1-1*), while a mutant affected in starch mobilization (*starch excess 1-1*, *sex1-1*) showed a slightly milder phenotype. However, galacturonic acid and cellulose contents were not significantly reduced in these genotypes (Fig. 4B). To examine whether reduced arabinose and galactose levels are caused by changes in soluble arabinogalactan proteins, we isolated water-soluble cell wall components of wild type, *pgm*, *adg1-1*, and *sex1-1* leaves. The soluble fraction was mainly composed of arabinose and galactose (indicative of arabinogalactan) and showed increased fucose and decreased arabinose levels in *pgm*, while no differences were observed in *adg1-1* and *sex1-1* compared with wild type (see Supplementary Fig. S1).

Next, we tested whether penetration resistance against *C. higginsianum* is also affected in *adg1-1* and *sex1-1* mutants. Consistent with the observed overall cell wall monosaccharide composition, the penetration frequency of *C. higginsianum* was similarly increased in *pgm* and *adg1-1*, and intermediate in *sex1-1*. In wild type plants, 20% of all appressoria had penetrated the leaf surface to form hyphae at 2 dpi, as compared with 64% in *pgm*, 72% in *adg1-1*, and 45% in *sex1-1* (Fig. 4C). Our observations thus suggest a causal link between impaired starch metabolism, altered cell wall composition and penetration frequency of *C. higginsianum*.

### Glycome analyses reveal significant alterations in cell wall glycan composition and extractability of starch-deficient *pgm* mutants

**Fig. 5.**
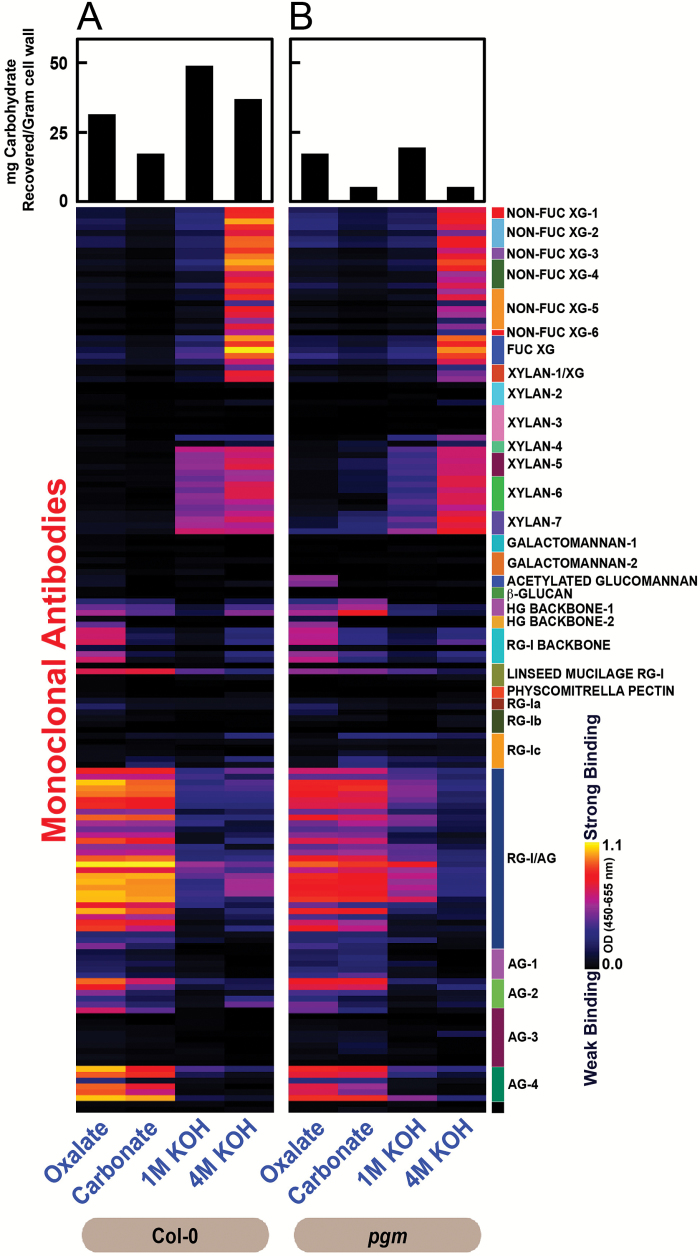
Glycome profiling of *pgm* mutants. Glycome profiling of sequential cell wall extracts prepared from rosette leaves of 5-week-old Col-0 (A) and *pgm* (B) plants. Samples represent pools of more than 50 fully expanded leaves collected in three independent experiments. Bar charts above the heat maps indicate the amount of carbohydrates recovered from cell wall preparations by sequential extraction with oxalate, carbonate, 1 M KOH and 4 M KOH. Extracts were analysed for abundance of cell wall glycan epitopes using 155 monoclonal antibodies (see Supplementary Table S1 and http://www.wallmabdb.net for details) whose glycan specificities are depicted on the right had side key. ELISA responses are illustrated in heat maps showing the relative binding strength of specific antibodies on a multi-color scale ranging from black (no binding) to bright yellow (strongest binding).

We performed glycome profiling analyses in order to elucidate differences in the extractability and overall composition of major non-cellulosic cell wall glycans between *pgm* and wild type leaves. Reduced amounts of carbohydrates were recovered from *pgm* cell wall preparations in comparison to the wild type in all extraction steps (Fig. 5, top bar charts). For ELISA analysis using cell wall glycan directed monoclonal antibodies (mAbs), equal amounts of recovered carbohydrates were coated, thus allowing for comparison of relative differences in mAb binding intensities between wild type and *pgm* extracts (Pattathil *et al.*, 2012).

Extractability of pectic rhamnogalacturonan-I/arabinogalactan epitopes (indicated by the binding of RG-I/AG and AG-4 groups of mAbs) was reduced in oxalate and carbonate extracts of *pgm* cell wall material, while the proportion of both unsubstituted and substituted xylan epitopes (those recognized by xylan-4 to -7 groups of mAbs) as well as of homogalacturonan (HG) backbone-1 and RG-I backbone epitopes was marginally increased in the *pgm* carbonate extract. Xylan extractability in 1 M KOH extract was reduced in the *pgm* cell wall preparation, while the abundance of RG-I/AG epitopes in this 1 M KOH extract of *pgm* was increased compared with wild type. Taken together, these differences may indicate that RG-I/AG epitopes are more tightly linked to the *pgm* cell wall matrix, while xylans are more loosely linked to the *pgm* cell wall matrix compared with wild type. Xyloglucan extractability patterns overall remained the same in *pgm* and wild type, with both non-fucosylated and fucosylated xyloglucans being abundant in 4 M KOH extracts. In addition, we observed an increased abundance of acetylated glucomannan epitopes in the oxalate extract of *pgm*.

In summary, these studies show considerable differences in the extractability of RG-I/AG, RG-I backbone, HG backbone, xylan and acetylated glucomannan epitopes from *pgm* cell walls, indicating significant overall alterations in the architecture of the *pgm* cell wall matrix.

### Comparative analysis of cell wall mutants indicates that pectin content is crucial for penetration resistance against *C. higginsianum* and biotrophic hyphae development

In order to gain insight into the relevance of different cell wall polymers for the susceptibility to *C. higginsianum*, we screened a number of cell wall mutants that (i) share particular alterations in cell wall composition with *pgm*, (ii) exhibit opposite changes compared with *pgm* or (iii) are likely to have altered cell wall stiffness. Loss of the putative arabinosyltransferase ARABINAN DEFICIENT 1 (ARAD1) and the UDP-D-xylose 4-epimerase MUR4 led to reduced arabinose content in leaf cell wall extracts (Burget and Reiter 1999; Burget *et al.*, 2003; Harholt *et al.*, 2006). Similarly, cell wall galactose levels were reduced in the galactosyltransferase mutant *galactan synthase 1* (*gals1*) (Liwanag *et al.*, 2012). Mutation of *MUR8* led to a reduced cell wall rhamnose content and glycosyl linkage analysis suggested a reduced RG-I content in *mur8-1* (Reiter *et al.*, 1997; Mertz *et al.*, 2012). A mutant of *MUR11/SUPRESSOR OF ACTIN 9* (*SAC9*) exhibits complex alterations in monosaccharide composition, with reduced rhamnose, fucose and xylose levels and increased mannose levels, likely caused by a constitutive stress response (Reiter *et al.*, 1997; Williams *et al.*, 2005; Austin *et al.*, 2011). Cell wall analysis of *pmr5 pmr6* double mutants indicated reduced fucose and increased arabinose, galactose and galacturonic acid content (Vogel *et al.*, 2004). Like in *pgm* mutants, the extractibility of xylan (xylan-4 to -7 mAbs) and pectin epitopes (HG and RG-I backbone mAbs) was increased in cell walls of *arabinoxylan pectin arabinogalactan protein 1* (*apap1*) mutants (Tan *et al.*, 2013). Finally, mutants of *PME3*, *PME35* and *PMEI6* have been analysed as alterations in DM and cell wall stiffness were expected in these mutants. Interaction phenotypes have previously been reported for *pmr5*, *pmr6*, *pme3* and *pme35*, in different pathosystems (Vogel *et al*., 2002, 2004; Raiola *et al.*, 2011; Bethke *et al.*, 2014).

**Fig. 6.**
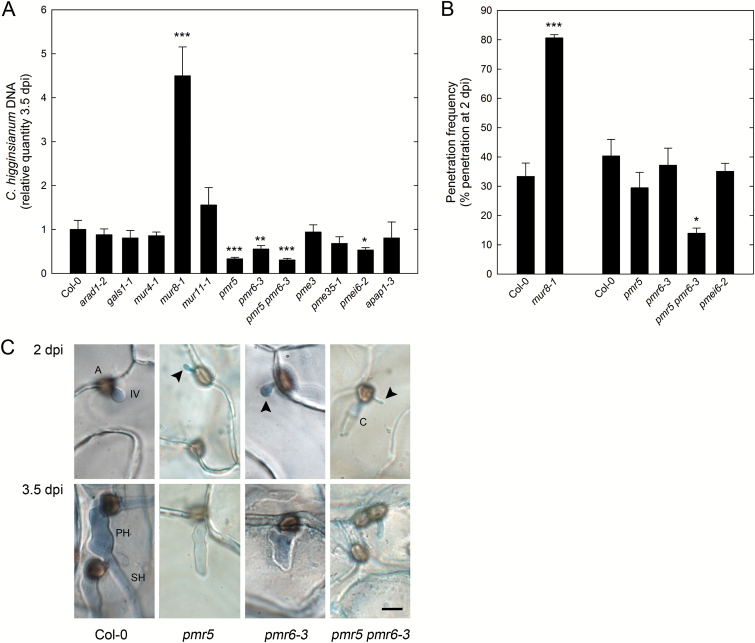
Analysis of fungal proliferation, penetration efficiency and hyphal morphology on Arabidopsis cell wall mutants. (A) The relative quantity of genomic *C. higginsianum* DNA in leaves of 5-week-old Col-0, *arad1-2*, *gals1-1*, *mur4-1*, *mur8-1*, *mur11-1*, *pmr5*, *pmr6-3*, *pmr5 pmr6-3*, *pme3*, *pme35-1*, *pmei6-2*, and *apap1-3* was determined by qPCR at 3.5 dpi. Values are means of three to four biological replicates measured in three technical replicates ±SE. For each replicate, fully expanded leaves of two infected plants were pooled. (B) Penetration frequency of *C. higginsianum* was analysed at 2 dpi in 5-week-old Col-0, *mur8-1*, *pmr5*, *pmr6-3*, *pmr5 pmr6-3*, and *pmei6-2* leaves. Values are means of three biological replicates ±SE. Per replicate, penetration success of hyphae formed from >250 appressoria on two leaves was scored. Asterisks indicate a significant difference to Col-0 (*P<0.05; **P<0.01; ***P<0.001; Student’s *t*-test). (C) Representative pictures of early fungal development in Col-0, *pmr5*, *pmr6-3*, and *pmr5 pmr6-3* leaves at 2 dpi and 3.5 dpi. C, conidium; A, appressorium; IV, infection vesicle; PH, primary hypha; SH, secondary hypha. Arrowheads point towards incompletely differentiated IVs. The bar represents 5 µm.

Of all mutants analysed, only *mur8-1* showed a strong increase in the amount of *C. higginsianum* DNA at 3.5 dpi compared with wild type (Fig. 6A). Increased fungal proliferation at this late interactions stage is not linked to a defect in major carbohydrate metabolism of *mur8-1* (see Supplementary Fig. S2 at *JXB* online). Reduced susceptibility was found for *pmr5*, *pmr6-3*, *pmr5 pmr6-3* and *pmei6-2* mutants. We therefore tested whether early establishment of *C. higginsianum* was also altered in these mutants. Penetration frequency was strongly increased in *mur8-1* and decreased in *pmr5 pmr6-3* (Fig. 6B). While penetration frequency was not affected in *pmr5* and *pmr6-3* single mutants, morphology of biotrophic hyphae formed in all *pmr* single and double mutants was severely altered (Fig. 6C). At 2 dpi, bulbous IVs were not observed in *pmr5* and *pmr5 pmr6-3*, while bulbous IVs were less frequent in *pmr6-3* than in wild type epidermal cells. During subsequent growth on *pmr* mutants, *C. higginsianum* hyphae either failed to branch and differentiate to SH, or IVs were shrunken. Thus, restricted fungal proliferation in *pmr5*, *pmr6-3*, and *pmr5 pmr6-3* at 3.5 dpi can be seen as a consequence of impaired development of biotrophic hyphae earlier in the interaction.

Altogether, these observations indicate a connection between altered pectin amount in *mur8-1*, *pmr5*, *pmr6-3*, and *pmr5 pmr6-3* with biotrophic establishment of *C. higginsianum*. In turn, our results suggest that differences in pectin content or structure might affect penetration resistance in *pgm*. Reduced arabinose and galactose levels in the *pgm* cell wall as well as reduced extractability of RG-I/AG epitopes indicated that arabinan and galactan side chains of RG-I might be altered in *pgm*. We thus digested crude cell wall preparations of *pgm*, *arad1-2*, *gals1-1* and wild type leaves with arabinanase and galactanase, to test whether enzymatic extractability of arabinose or galactose is altered in *pgm* compared with these lines. While an arabinanase digestion liberated 19.4 ± 3.7% of the total arabinose in *pgm* samples, only 9.3 ± 4.6% and 6.7 ± 3.4% of the arabinose residues could be released in wild type and *arad1-2* cell wall preparations, respectively. This significant difference (*P*<0.01, Student’s *t*-test) indicates a structural difference between *pgm* and *arad1-2* cell walls. In contrast, a galactanase digest of crude cell wall material did not reveal any difference between *pgm*, *gals1-1*, and wild type.

## Discussion

In the present study, we have observed that penetration frequency and establishment of *C. higginsianum* hyphae were enhanced in leaves of the starch-deficient mutants *pgm* and *adg1-1* and the starch-excess mutant *sex1-1*, which is hampered in starch mobilization. While these mutants suffer from nocturnal carbon limitation, the accelerated fungal development during the early interaction phase with these genotypes cannot be explained by the previously reported negative effect of reduced carbon availability on induced defense responses (Engelsdorf *et al.*, 2013). Both salicylic acid and camalexin start to accumulate after successful penetration of host cells, when the establishment of biotrophic hyphae is already completed (Engelsdorf *et al.*, 2013). Therefore, either preexisting (structural) defense, induced preinvasion defense or immediate post-penetration defense must account for the enhanced establishment of *C. higginsianum* in Arabidopsis genotypes with impaired starch metabolism.

The data obtained for the *pgm* mutant indicate that the cuticle composition and the diminished production of indolic glucosinolates (IGs), both major determinants of pre-invasion and non-host resistance (e.g. Bednarek *et al.*, 2009; Sanchez-Vallet *et al.*, 2010, Schlaeppi *et al.*, 2010; Hansjakob *et al.*, 2011; Uppalapati *et al.*, 2012; Hiruma *et al.*, 2013), do not play a major role for the earlier establishment of *C. higginsianum* on host leaves with impaired starch metabolism.

### Regulation of indolic glucosinolate biosynthesis might contribute to postinvasion resistance against *C. higginsianum*

The IG 4MOI3M is supposed to serve as a substrate for the atypical myrosinase PEN2. The PEN2 reaction product is required for penetration resistance against non-adapted powdery mildews (Bednarek *et al.*, 2009; Clay *et al.*, 2009). The resistance of *pen2-2* was significantly diminished toward two *C. higginsianum* pathogenicity mutants, but not toward *C. higginsianum* wild type (Huser *et al.*, 2009). Therefore, the contribution of IGs to resistance against *C. higginsianum* seemed to be of a quantitative nature. We observed that 4MOI3M accumulated during leaf infections with *C. higginsianum*. I3M and 4MOI3M contents were significantly reduced in *pgm* leaves after infection compared with wild type, suggesting that carbon limitation in *pgm* disfavors carbon flux to glucosinolates. Nonetheless, a reduced capacity for IG production in the mutants *hig1-1* and *myb34 myb51* (Gigolashvili *et al.*, 2007; Frerigmann and Gigolashvili, 2014) did not affect penetration frequency of *C. higginsianum*. Therefore, we can exclude that the reduced accumulation of I3M and 4MOI3M contributes to the expedited establishment of *C. higginsianum* in the *pgm* mutant.

At the time of extensive fungal proliferation during the necrotrophic growth phase, genotypes with both a reduction (*hig1-1*, *myb34/51*) and an increase in IG production (*HIG1-1D*) allowed for a more than three-fold increase in *C. higginsianum* colonization. However, this interaction phenotype may not exclusively be accounted for by altered IG production. The R2R3-MYB transcription factors MYB34 and MYB51 induce the cytochrome P450 monooxygenase isoforms CYP79B2 and CYP79B3, which catalyse the synthesis of indole-3-acetaldoxime (IAOx). IAOx formation from tryptophan is the committed step not only toward IG synthesis, but also toward the production of the major Arabidopsis phytoalexin camalexin, which is believed to disturb cellular integrity of fungal pathogens (Frerigmann *et al.*, 2015). CYP79B2/B3 induction was almost absent in *myb34/51/122* triple mutants, which did not differ from *myb34/51* in indolic glucosinolate accumulation (Frerigmann and Gigolashvili, 2014). These findings suggest a dramatic reduction of IAOx production in *myb34/51* double mutants as well. However, camalexin contents were significantly altered neither in *hig1-1*, *myb34/51*, and *HIG1-1D* after infection with *C. higginsianum* (not shown) nor in *myb34/51/122* after infection with *Plectosphaerella cucumerina* (Frerigmann *et al.*, 2016). Given that cruciferous species can derive other indolic phytoalexins from IAOx (Hagemeier *et al.*, 2001; Pedras *et al.*, 2001), it cannot be excluded that manipulation of IAOx production in *hig1-1*, *myb34/51*, and *HIG1-1D* affects the synthesis of indolic phytoalexins, e.g. like indole-3-carbaldehyde or indole-3-carboxylic acid derivatives (Hagemeier *et al.*, 2001), which might play an important role for the defense against necrotrophic colonization by *C. higginsianum*.

### Starch deficiency influences cell wall metabolism and composition

Due to the lack of starch, the transitory carbon storage capacity of the Arabidopsis *pgm* mutant is reduced by 66% in our growth conditions (Engelsdorf *et al.*, 2013), making the mutant suffer from periodic carbon shortage towards the end of each dark phase, when the vacuolar sucrose reserves are depleted (Gibon *et al.*, 2004; Engelsdorf *et al.*, 2013). In transcriptome analyses of *pgm* by Thimm *et al.* (2004) and Bläsing *et al*. (2005), genes associated with cell wall metabolism were two-fold overrepresented among the downregulated genes at the end of the dark period, suggesting that cell wall metabolism is altered upon carbon shortage in *pgm*. The data presented here show a consistent reduction of arabinose and galactose levels and an increase in fucose, xylose and mannose levels in the cell walls of two mutants affected in starch biosynthesis (*pgm*, *adg1-1*) and one mutant affected in starch mobilization (*sex1-1*). Overall extractability of carbohydrates from *pgm* cell walls was strongly reduced. Particularly pectic epitopes (RG-I/AG and AG-4 groups of mAbs) showed reduced extractability, suggesting tighter linkage to other cell walls polymers. In turn, xylan epitopes (xylan-4 to -7 groups of mAbs) could be extracted in milder conditions, suggesting weaker linkage to other cell wall polymers. Taken together, our data indicate that pectic arabinan and galactan content is reduced in *pgm* cell walls, while xylan content is increased. The differences observed in pectin composition can either be explained by reduced arabinan and galactan synthesis or increased pectin degradation. Interestingly, an increased abundance of acetylated-glucomannan epitopes (recognized by mAbs, CCRC-M169 and CCRC-M170) was observed in the oxalate extract from *pgm* cell walls. However, additional studies are required to fully understand what structural difference(s) in *pgm* cell walls cause this increased abundance of these epitopes.

Pectic polysaccharides serve as carbohydrate storage in cotyledons of leguminous plants (Buckeridge *et al.*, 2005; Gronwald *et al.*, 2009). During mobilization of cell wall storage polysaccharides (CWSPs) in soybean cotyledons, cell wall galactose and arabinose content decrease by more than 85% (Gronwald *et al.*, 2009), while galactan chains were shown to be the predominant CWSP in lupin cotyledons (Buckeridge *et al.*, 2005). A study by Lee *et al.* (2007) demonstrated that after prolonged carbon starvation in detached Arabidopsis leaves for 2 d, the amount of all monosaccharides in pectin and hemicelluloses was substantially reduced by at least 30%, indicating an excessive mobilization of carbon from the cell wall under these extreme conditions. It seems reasonable to speculate that the degradation of cell wall polymers is restrained under *in vivo* conditions in intact plants. Released galactose moieties activated by galactokinase (GALK) and UDP-sugar pyrophosphorylase (USP) can be interconverted into UDP-glucose by UDP-glucose 4-epimerases (UGEs) to fuel sucrose biosynthesis in times of need (Reiter, 2008; Geserick and Tenhaken, 2013). Five UGE genes are present in Arabidopsis, of which UGE1 and UGE3 are supposed to be involved in cell wall catabolism (Barber *et al.*, 2006). Analysis of recombinant Arabidopsis UGEs in *E. coli* revealed that UGE1 and UGE3 additionally have high UDP-xylose 4-epimerase activity (Kotake *et al.*, 2009), being able to also interconvert UDP-arabinose and UDP-xylose. Similar to galactose, arabinose moieties can be activated by arabinokinase (ARA) and USP and converted into UDP-xylose by UGEs and UDP-xylose 4-epimerase (MUR4) (Reiter, 2008).

Compared with the wild type, *GALK2*, *ARA1*, *UGE1*, and *UGE3* were upregulated in *pgm* mutants during the second half of the dark phase and Col-0 rosettes subjected to an extended night (see Supplementary Fig. S3; Usadel *et al.*, 2008). In addition, accessibility of arabinose monomers to arabinanase digest was elevated in *pgm* cell wall preparations compared with wild type and *arad1-2* mutants. Recently, Behmüller *et al*. (2016) suggested that ARA1 is involved in sugar signaling during arabinose feeding, thus potentially sensing flux through the salvage pathway. On the other hand, we examined gene expression levels of galactosyltransferase (*GALS1–GALS3*) and putative arabinosyltransferase (*ARAD1* and *ARAD2*) genes, which are supposed to be involved in galactan and arabinan biosynthesis, in *pgm* mutants during the second half of the dark phase and Col-0 rosettes subjected to an extended night. *GALS1* and *GALS3* expression was consistently downregulated in both data sets, suggesting that galactan synthesis is attenuated upon carbon shortage, whereas *ARAD1* and *ARAD2* expression was unchanged compared with the respective controls (Supplementary Fig. S3; Usadel *et al.*, 2008).

In summary, the available data indicate that galactose and arabinose moieties are mobilized and salvaged from *pgm* pectin during periods of nocturnal starvation while galactan synthesis is attenuated.

### Pectin levels affect penetration frequency of *C. higginsianum* in Arabidopsis leaves

To test whether specific alterations in *pgm* cell walls account for the impaired penetration resistance against *C. higginsianum*, we screened a number of cell wall mutants for susceptibility phenotypes. As Arabidopsis mutants with a specific increase in leaf cell wall xylans have not been identified to our knowledge, we could not test the contribution of xylans in *pgm*. Susceptibility of the *arad1-2* mutant with reduced arabinan (Harholt *et al.*, 2006) and the *gals1-1* mutant with diminished galactan side chains (Liwanag *et al.*, 2012) was comparable to the wild type. Thus, we can rule out that increased *pgm* susceptibility is caused by simple deficiencies in either polymer. Susceptibility was also unaffected in *mur4-1*, *mur11-1*, *pme3*, *pme35-1*, and *apap1-3* mutants, indicating that specific cell wall alterations in these mutants do not affect susceptibility to *C. higginsianum* either. While fungal DNA amount was reduced in *pmei6-2* mutants at 3.5 dpi, penetration resistance was not affected, suggesting restricted necrotrophic growth in this mutant. Notably, the *mur8-1* mutant, which has a reduced RG-I content in leaf cell walls (Mertz *et al.*, 2012), was both hypersusceptible toward *C. higginsianum* and showed strongly impaired penetration resistance. Since the pectic polymer RG-I is rich in both arabinan and galactan side chains, it is tempting to assume that altered RG-I composition contributes to reduced penetration resistance in *pgm* as well.

On the other hand, *pmr5*, *pmr6-3*, and *pmr5 pmr6-3* mutants, which exhibit increased pectin content and altered pectin modification of epidermal cell walls (Vogel *et al*., 2002, 2004), showed increased resistance toward *C. higginsianum*. While penetration resistance was not affected in *pmr* single mutants, penetration frequency in the double mutant was reduced to 35% of wild type. Post-invasive differentiation of infection vesicles and biotrophic hyphae was severely hampered in both *pmr* single and *pmr* double mutants. Similarly growth and integrity of *E. cichoracearum* epiphytic hyphae were also affected in these *pmr* mutants (Vogel *et al*., 2002, 2004). Although a partial resistance of *pmr6* toward *C. higginsianum* had been reported previously (O’Connell and Panstruga, 2006), we have now shown that establishment of *C. higginsianum* is impaired on both *pmr5* and *pmr6* single and double mutants because of impaired hyphal expansion and differentiation. Increased pectin content in *pmr* mutants may simply provide a higher mechanical resistance to the invagination of the plasma membrane during formation of infection vesicles. Alternatively, an altered matrix between host and fungal cells might affect the perception of host signals by the fungus or restrict accessibility of nutrients to the fungus.

Taken together, our data suggest that Arabidopsis pectin content and composition influence both penetration by *C. higginsianum* and normal expansion of biotrophic *C. higginsianum* hyphae in host epidermal cells. Our data also strongly imply that enhanced penetration frequency of *C. higginsianum* in mutants with reduced starch turnover is caused by altered cell wall composition. As judged from our data, changes in pectin content or composition seem to be most relevant for altered penetration in these mutants.

## Supplementary Material

Supplementary Data
