# Supplementary material for: Cell wall composition and penetration resistance against the fungal pathogen Colletotrichum higginsianum are affected by impaired starch turnover in Arabidopsis mutants
Source: J Exp Bot. 2016 Dec 12;68(3):701–13. doi: 10.1093/jxb/erw434 (PMC5441917; doi:10.1093/jxb/erw434)
Supplement: Supplementary Data [file erw434_Supplementary_Data.zip › supplementary_figures_S1_S3_Table_S1.pdf]

Supplementary Material

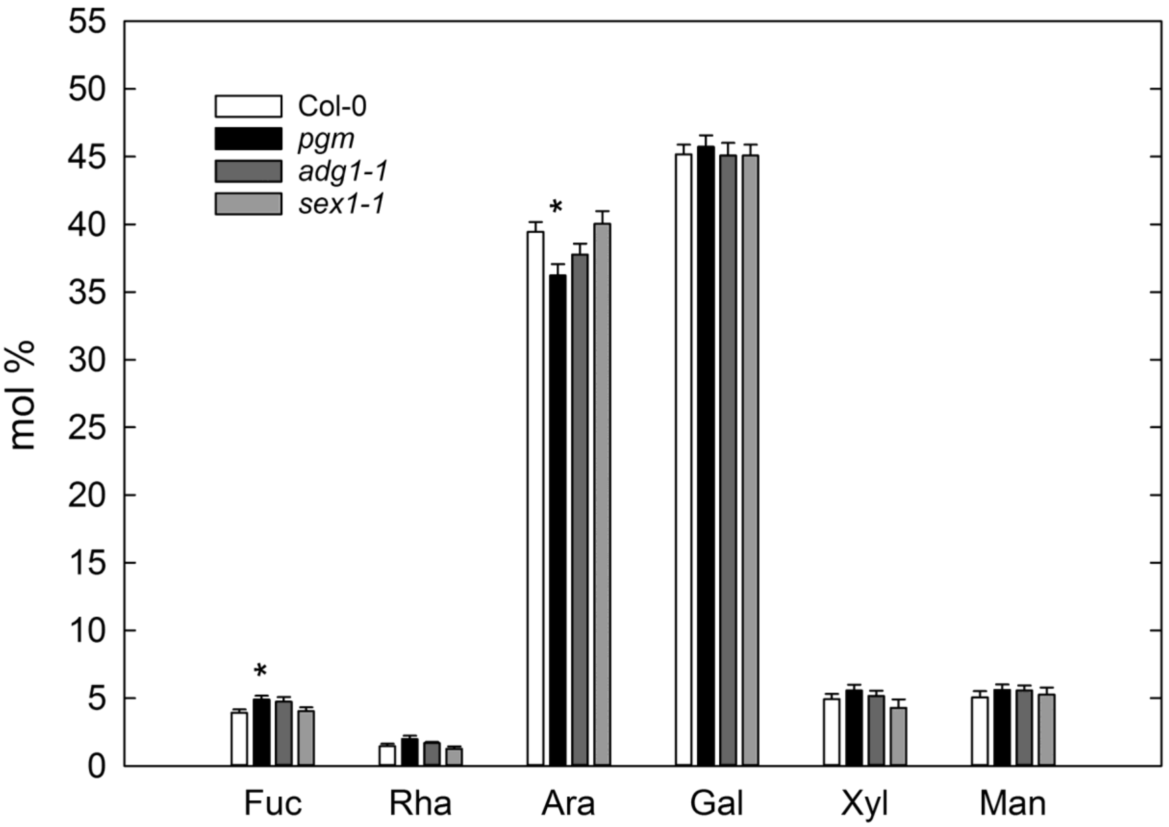

Figure S1. Monosaccharide composition of water-soluble cell wall polymers extracted from Arabidopsis mutants with impaired starch metabolism.

Water-soluble polymers were extracted from cell wall preparations prior to analysis of the relative monosaccharide composition in 5-week-old rosette leaves of Col-0, *pgm*, *adg1-1* and *sex1-1*. Fuc, fucose; Rha, rhamnose; Ara, arabinose; Gal, galactose; Xyl, xylose; Man, mannose. Col-0, white bars; *pgm*, black bars; *adg1-1*, dark grey bars; *sex1-1*, light grey bars. Values are means ± SE (n=6). Asterisks indicate a significant difference to Col-0 (*P < 0.05; **P < 0.01; ***P < 0.001; Student's *t*-test).

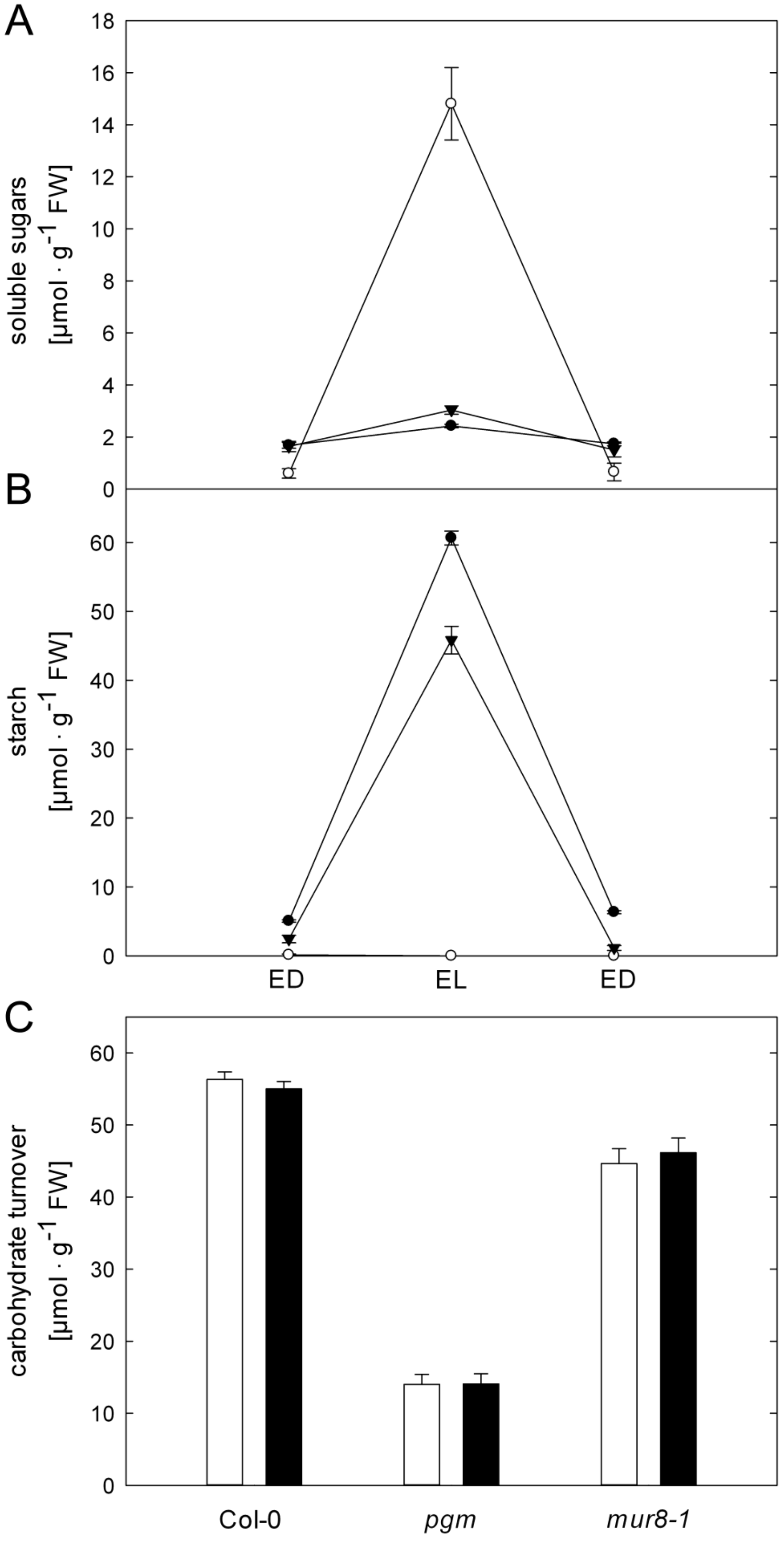

A
soluble sugars
[µmol · g-1 FW]
B
starch
[µmol · g-1 FW]
ED
EL
ED
C
carbohydrate turnover
[µmol · g-1 FW]
Col-0
pgm
mur8-1

Figure S2. Diurnal accumulation and turnover of major carbohydrates in *mur8-1.*

(A) Soluble sugar and **(B)** starch content of 5-week-old Col-0, *pgm* and *mur8-1* leaves were monitored at the end of the dark phase (ED) and at the end of the light phase (EL) under 12h/12h L/D conditions. Col-0, black circles; *pgm*, white circles; *mur8-1*; black triangles. Values are means of four biological replicates ± SE.

(C) The diurnal carbohydrate turnover was calculated from the data depicted in A and B. Carbohydrate accumulation, white bars; carbohydrate degradation, black bars. Values are means of four biological replicates ± SE.

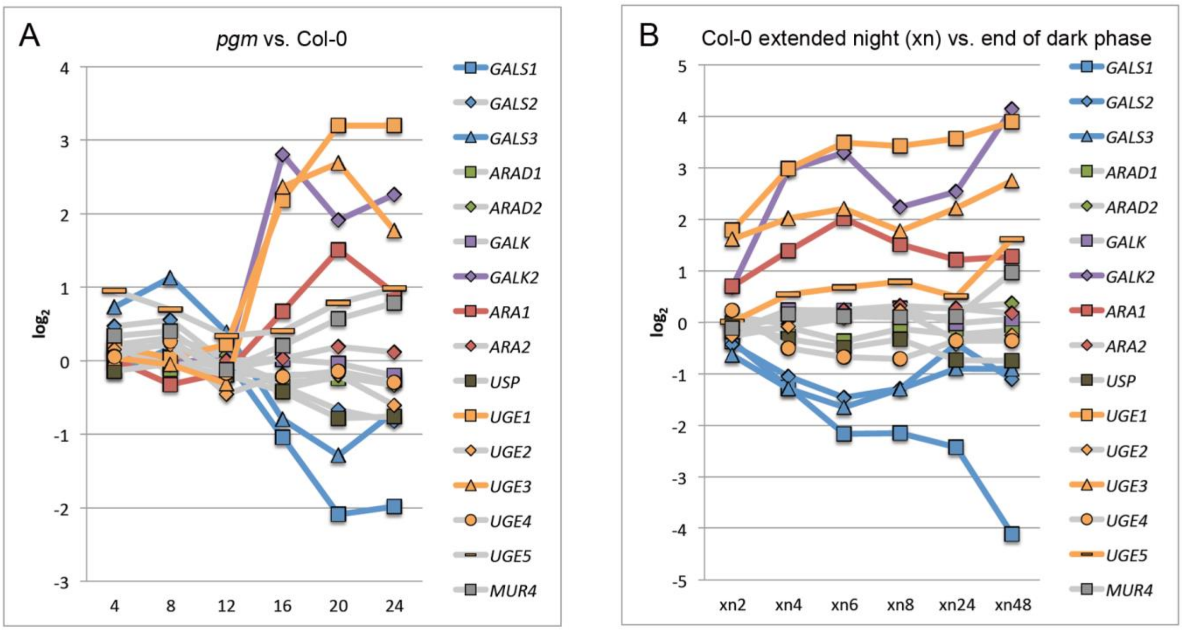

Figure S3. Diurnal expression of genes involved in the metabolism of UDP-galactose and UDP-arabinose in *pgm* mutant leaves and Col-0 wild type leaves during an extended night.

(A) Relative gene expression in *pgm* rosette leaves compared to Col-0 4, 8, 12, 16, 20 and 24 h after start of the light phase in a 12h/12h (L/D) cycle.

(B) Relative gene expression in Col-0 rosette leaves after 2, 4, 6, 8, 24 and 48 h of extended night compared to the end of the regular dark phase.

Microarray data were taken from Usadel et al. (2008). *GALS, GALACTAN SYNTHASE; ARAD, ARABINAN DEFICIENT; GALK, GALACTOKINASE; ARA, ARABINOKINASE; USP, UDP-SUGAR PYROPHOSPHORYLASE; UGE, UDP-D-GLUCOSE 4-EPIMERASE; MUR, MURUS*. Symbols of genes from the same family are coloured alike. For genes with expression changes smaller than 2-fold compared to the controls at all time points, lines are drawn in grey.

Table S1. Detailed list of cell wall glycan-directed monoclonal antibodies (mAbs) used for glycome profiling analyses. (see next pages)

The antibody grouping is based on hierarchical clustering of ELISA data generated in a screen of all mAbs against a comprehensive panel of plant polysaccharide preparations (Pattathil et al., 2010; 2012); mAbs cluster according to the predominant polysaccharides that they recognize. The majority of listings link to the Wall*Mab*DB plant cell wall monoclonal antibody database (http://www.wallmabdb.net) that provides detailed descriptions of each mAb, including immunogen, antibody isotype, epitope structure (to the extent known), supplier information, and related citations.

**Glycan Group Recognized**	**mAb Name**
Non-Fucosylated Xyloglucan-1	CCRC-M95
	CCRC-M101
Non-Fucosylated Xyloglucan-2	CCRC-M104
	CCRC-M89
	CCRC-M93
	CCRC-M87
	CCRC-M88
Non-Fucosylated Xyloglucan-3	CCRC-M100
	CCRC-M103
Non-Fucosylated Xyloglucan-4	CCRC-M58
	CCRC-M86
	CCRC-M55
	CCRC-M52
	CCRC-M99
Non-Fucosylated Xyloglucan-5	CCRC-M54
	CCRC-M48
	CCRC-M49
	CCRC-M96
	CCRC-M50
	CCRC-M51
	CCRC-M53
Non-Fucosylated Xyloglucan-6	CCRC-M57
Fucosylated Xyloglucan	CCRC-M102
	CCRC-M39
	CCRC-M106
	CCRC-M84
	CCRC-M1
Xylan-1/XG	CCRC-M111
	CCRC-M108
	CCRC-M109

Xylan-2	CCRC-M119
	CCRC-M115
	CCRC-M110
	CCRC-M105
Xylan-3	CCRC-M117
	CCRC-M113
	CCRC-M120
	CCRC-M118
	CCRC-M116
	CCRC-M114
Xylan-4	CCRC-M154
	CCRC-M150
Xylan-5	CCRC-M144
	CCRC-M146
	CCRC-M145
	CCRC-M155
Xylan-6	CCRC-M153
	CCRC-M151
	CCRC-M148
	CCRC-M140
	CCRC-M139
	CCRC-M138
Xylan-7	CCRC-M160
	CCRC-M137
	CCRC-M152
	CCRC-M149
Galactomannan-1	CCRC-M75
	CCRC-M70
	CCRC-M74
Galactomannan-2	CCRC-M166
	CCRC-M168
	CCRC-M174
	CCRC-M175

Glucomannan	CCRC-M169<br>CCRC-M170
β-Glucan	LAMP<br>BG1
HG Backbone-1	CCRC-M131<br>CCRC-M38<br>JIM5
HG Backbone-2	JIM136<br>JIM7
RG-I Backbone	CCRC-M69<br>CCRC-M35<br>CCRC-M36<br>CCRC-M14<br>CCRC-M129<br>CCRC-M72
Linseed Mucilage RG-I	JIM3<br>CCRC-M40<br>CCRC-M161<br>CCRC-M164
Physcomitrella Pectin	CCRC-M98<br>CCRC-M94
RG-Ia	CCRC-M5<br>CCRC-M2
RG-Ib	JIM137<br>JIM101<br>CCRC-M61<br>CCRC-M30
RG-Ic	CCRC-M23<br>CCRC-M17<br>CCRC-M19<br>CCRC-M18<br>CCRC-M56<br>CCRC-M16

RG-I/Arabinogalactan	CCRC-M60
	CCRC-M41
	CCRC-M80
	CCRC-M79
	CCRC-M44
	CCRC-M33
	CCRC-M32
	CCRC-M13
	CCRC-M42
	CCRC-M24
	CCRC-M12
	CCRC-M7
	CCRC-M77
	CCRC-M25
	CCRC-M9
	CCRC-M128
	CCRC-M126
	CCRC-M134
	CCRC-M125
	CCRC-M123
	CCRC-M122
	CCRC-M121
	CCRC-M112
	CCRC-M21
	JIM131
	CCRC-M22
	JIM132
	JIM1
	CCRC-M15
	CCRC-M8
	JIM16
Arabinogalactan-1	JIM93
	JIM94
	JIM11
	MAC204
	JIM20

Arabinogalactan-2	JIM14
	JIM19
	JIM12
	CCRC-M133
	CCRC-M107
Arabinogalactan-3	JIM4
	CCRC-M31
	JIM17
	CCRC-M26
	JIM15
	JIM8
	CCRC-M85
	CCRC-M81
	MAC266
	PN 16.4B4
Arabinogalactan-4	MAC207
	JIM133
	JIM13
	CCRC-M92
	CCRC-M91
	CCRC-M78
Unidentified	MAC265
	CCRC-M97
